# Spatial and Temporal Characteristics of 2014 Dengue Outbreak in Guangdong, China

**DOI:** 10.1038/s41598-018-19168-6

**Published:** 2018-02-05

**Authors:** Mattia Sanna, Jianyong Wu, Yanshan Zhu, Zhicong Yang, Jiahai Lu, Ying-Hen Hsieh

**Affiliations:** 1 Department of Public Health, China Medical University, Taichung, Taiwan; 2 School of Public Health, Sun Yat-Sen University, Guangzhou, China; 3 Key Laboratory for Tropical Disease Control of Ministry of Education, Sun Yat-Sen University, Guangzhou, China; 4 One Health Center of Excellence for Research &Training, Sun Yat-Sen University, Guangzhou, China; 5 Zhongshan Research Institute, School of Public Health, Sun Yat-Sen University, Zhongshan, China; 6 Guangzhou Center for Disease Control and Prevention, Guangzhou, China; 7 Center for Infectious Disease Education and Research, China Medical University, Taichung, Taiwan

## Abstract

The record-breaking number of dengue cases reported in Guangdong, China in 2014 has been topic for many studies. However, the spatial and temporal characteristics of this unexpectedly explosive outbreak are still poorly understood. We adopt an integrated approach to ascertain the spatial-temporal progression of the outbreak in each city in Guangdong as well as in each district in Guangzhou, where the majority of cases occurred. We utilize the Richards model, which determines the waves of reported cases at each location and identifies the turning point for each wave, in combination with a spatial association analysis conducted by computing the standardized G* statistic that measures the degree of spatial autocorrelation of a set of geo-referenced data from a local perspective. We found that Yuexiu district in Guangzhou was the initial hot spot for the outbreak, subsequently spreading to its neighboring districts in Guangzhou and other cities in Guangdong province. Hospital isolation of cases during early stage of outbreak in neighboring Zhongshan (in effort to prevent disease transmission to the vectors) might have played an important role in the timely mitigation of the disease. Integration of modeling approach and spatial association analysis allows us to pinpoint waves that spread the disease to communities beyond the borders of the initially affected regions.

## Introduction

Dengue fever (DF) is an infectious disease, currently affecting most of the regions lying within the tropical and subtropical belts. It is caused by dengue virus, and is vectored by mosquitoes belonging to the genus *Aedes*, which thrive in hot and humid climates. Its worldwide quick spread in the last decades is unprecedented and worrisome, to the extent that World Health Organization has recently included DF in the list of the potential public health emergencies of international concern^1^.

Currently, the highest-risk area for DF infection is the Asia-Pacific region, which is home to 75% of the world population exposed to dengue virus^2^. Located in this area, the southern region of China had more than 650,000 reported cases (with 610 fatalities) from 1978 to 2008. According to a recent study^3^, this situation seems to forebode the transformation of DF from an imported to an endemic disease. No effective dengue virus vaccine is currently available in China. Hence the most widely used intervention measures are suppressing vector population and blocking chain of disease transmission (e.g. home isolation of mild cases), as implemented by Guangdong Provincial Health and Family Planning Commission^4^.

In the last decades, the most severe outbreak was recorded in 2014, with 47,056 laboratory-confirmed infections^5^, mostly reported in Guangdong province (>95%). Here, unlike in many other dengue risk regions, the dominant mosquito species is *Aedes albopictus*^6^. Moreover, in its capital city, Guangzhou, there is an intense flow of people to and from Southeast Asia^7^, which increases the probability of imported cases. The 2014 record-breaking number of cases has drawn the attention of many researchers, leading to several (and quite different) explanations for such an unusual event. For examples, Cheng *et al*.^8^ identifies the date of the first imported case and the abnormal rainfall in May and August as the main factors. Li *et al*.^9^ suggests that there had been some delay in intervention strategy and further stresses the important role of asymptomatic infections. Zhu *et al*.^10^ highlights the key role played by population density and mobility. Lin *et al*.^11^ concludes that DF had become endemic in Guangdong province. Tian *et al*.^12^ detects an interesting association with the increased amount of surface water area in 2014.

It has been suggested that, although some advances have been made in the last decades, simple methods for evaluating dengue epidemiology over time and space are still much needed by public health authorities, since the tools developed in recent decades are rarely used due to their complexity and extensive data requirements^13^.

Within this context, the recent rapid development of Geographic Information Systems (GIS) has been gaining more and more attention, since it provides epidemiologists with new tools for studying the spatial component of an infectious disease spread. In particular, spatial clustering analysis is nowadays largely utilized for disease surveillance^14^, due to its ability to identify agglomerations of homogeneous locations, and to detect anomalies in the spatial distribution of a variable. For instance, Thanh Toan *et al*.^15^ adopted this approach to assess the geographic expansion of dengue transmission in Hanoi (Vietnam); Acharya *et al*.^16^ were able to map for the first time the spatial-temporal distribution of dengue at district level in Nepal; Espinosa *et al*.^17^ observed that their results could be utilized by public health professional to target high-risk areas for infection in Argentina. For a review of dengue models in literature focusing on climate change and socio-environmental factors in the Asia-Pacific region, please see Banu *et al*.^18^.

In general, spatiotemporal mapping is a powerful technique, with which users can easily and meaningfully display the variations over time and space of a specific variable. As a consequence, temporal and spatial evolution of an outbreak can be promptly assessed, providing crucial information to the planning of prevention and control programs^19^.

The aim of this research is to examine the spatial-temporal characteristics of the 2014 outbreak in Guangdong province, coupling a mathematical modeling approach, able to describe the temporal dynamic of the epidemic, with a spatial association analysis, for identifying hot spots for infection. The information supplied by the two methods is to some extent complementary, thus providing a clearer picture of the spatial-temporal evolution of the epidemic.

## Results

### The Richards model

**Figure 1:**
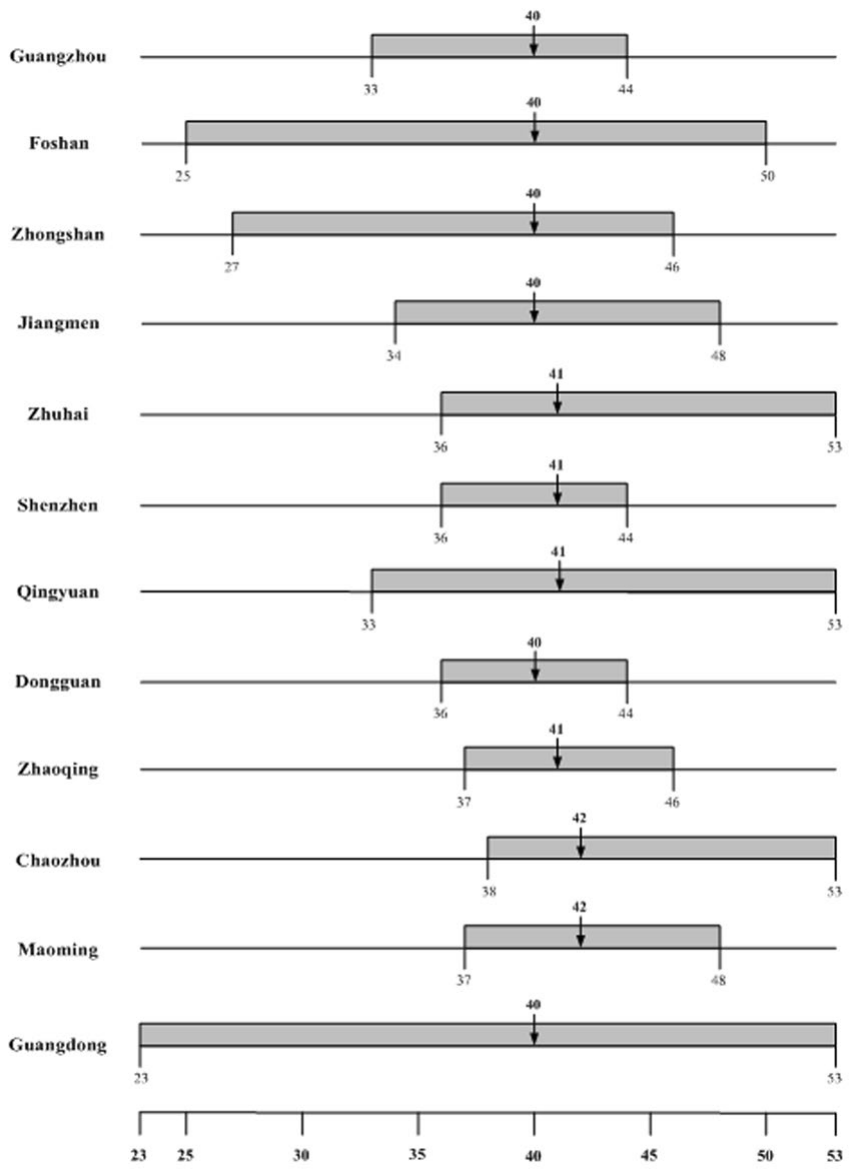
Timeline for dengue outbreak by city in Guangdong province.

**Figure 2:**
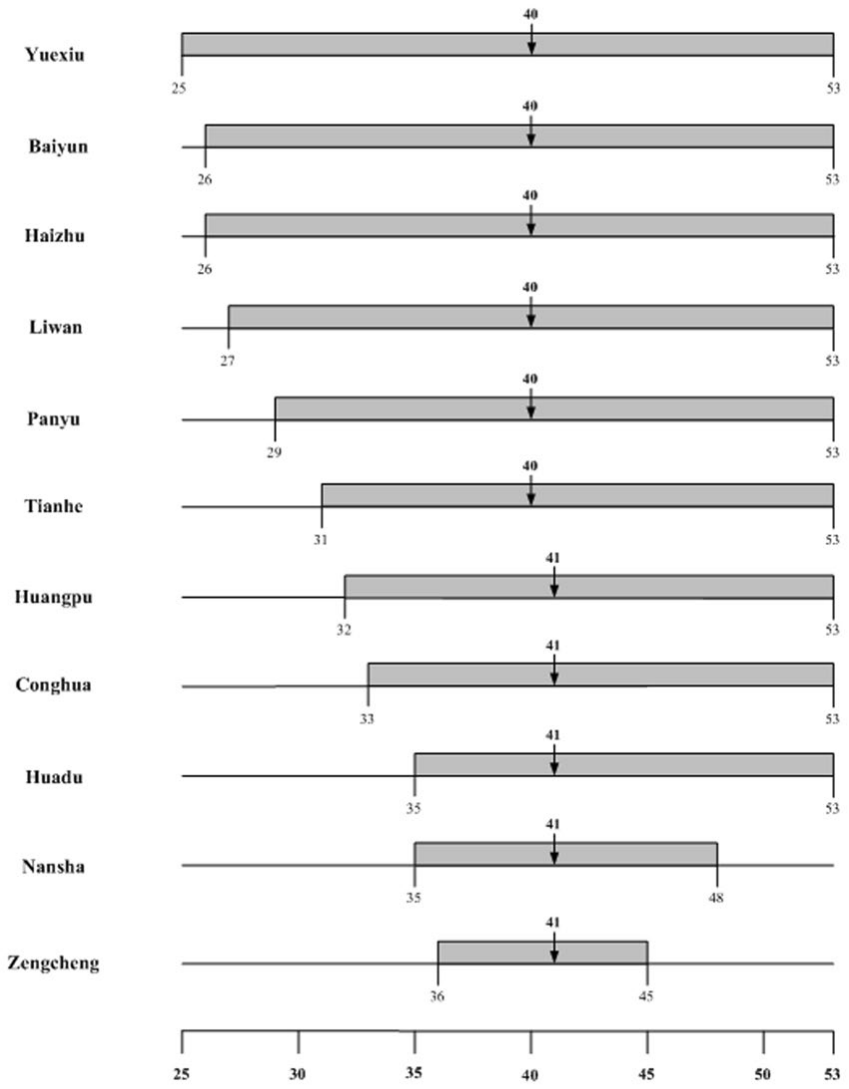
Timeline for dengue outbreak by district in Guangzhou city.

**Figure 3:**
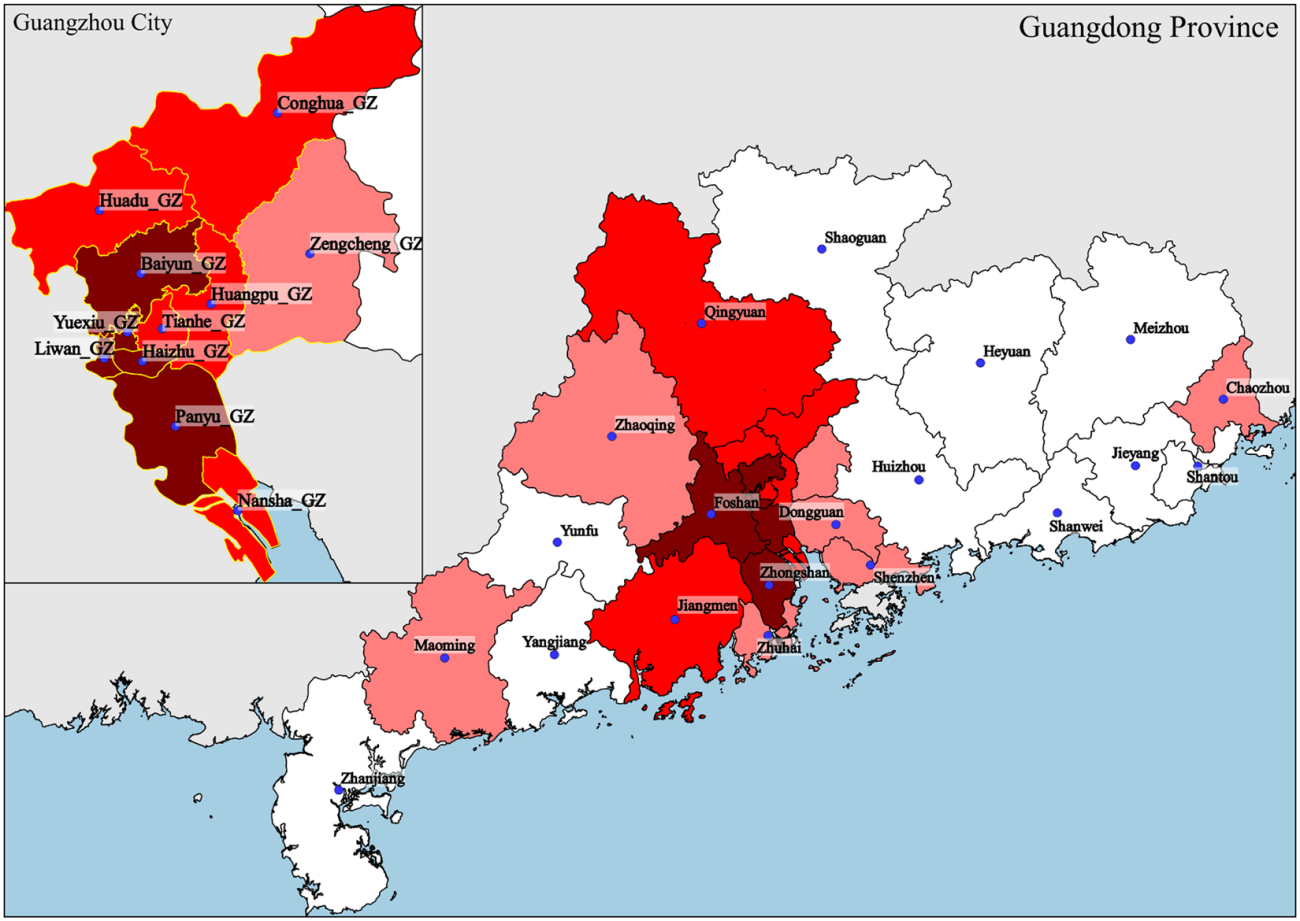
Map of spatial-temporal spread of dengue in Guangdong in 2014 by starting time of a wave of infections. Darker shade of red denotes earlier start of a wave. Dark red color indicates a starting time between week 25 and week 29, light red between week 31 and week 35, and pink between week 36 and week 38. Figure 3 was created with the Open Source software QGIS (version 2.16.3 - http://www.qgis.org/en/site/).

We fit the 2014 weekly confirmed dengue case data to the Richards model^20^, resulting in exactly one wave of cases for all the 11 districts in Guangzhou city, and for 11 of the 21 cities in Guangdong province (Tables 1 and 2). The timelines of the waves are given in Figs 1 and 2, while a map illustrating the spatial-temporal shift is displayed in Fig. 3, with the starting time of waves of cases in each district/city grouped in different shades of red.

**Table 1:** Summary table for estimated model parameters by fitting 2014 Guangdong cities weekly confirmed dengue data to the Richards model.

City	Time interval	Growth rate r (95% CI)	Case number K (95% CI)	Turning point (week)
Guangzhou	W33~W44	0.56 (0.51, 0.61)	36,342 (35,753, 36,932)	40
Foshan	W25~W50	0.86 (0.73, 0.98)	3550 (3,529, 3,571)	40
Zhongshan	W27~W46	0.69 (0.62, 0.76)	673 (666, 679)	40
Jiangmen	W34~W48	0.94 (0.47, 1.41)	590 (577, 604)	40
Zhuhai	W36~W53	0.94 (0.61, 1.26)	508 (502, 513)	41 (40.06)
Shenzhen	W36~W44	1.39 (0.72, 2.06)	385 (367, 403)	41
Qingyuan	W33~W53	1.29 (0.57, 2.02)	297 (293, 301)	41
Dongguan	W36~W44	1.22 (0.90, 1.55)	267 (262, 273)	40
Zhaoqing	W37~W46	2.38 (1.96, 2.80)	275 (274, 276)	41 (40.04)
Chaozhou	W38~W53	1.00 (0.34, 1.65)	137 (134, 140)	42
Maoming	W37~W48	0.72 (0.40, 1.03)	91 (88, 94)	42
Guangdong	W23~W53	0.63 (0.58, 0.68)	44,984 (44,812, 45,157)	40

**Table 2:** Summary table for estimated model parameters by fitting 2014 Guangzhou districts weekly confirmed dengue data to the Richards model.

District	Time interval	Growth rate r (95% CI)	Case number K (95% CI)	Turning point (week)
Yuexiu	W25~W53	0.35 (0.33, 0.36)	4,779 (4,761, 4,798)	40
Baiyun	W26~W53	0.87 (0.76, 0.97)	11,803 (11,751, 11,855)	40
Haizhu	W26~W53	0.65 (0.62, 0.68)	5,984 (5,970, 5,998)	40
Liwan	W27~W53	0.63 (0.56, 0.69)	4,452 (4,429, 4,474)	40
Panyu	W29~W53	0.69 (0.64, 0.74)	3,533 (3,522, 3,544)	40
Tianhe	W31~W53	0.75 (0.66, 0.83)	3,418 (3,401, 3,436)	40
Huangpu	W32~W53	0.71 (0.63, 0.78)	1,804 (1,795, 1,814)	41
Conghua	W33~W53	1.69 (0.89, 2.50)	105 (104, 106)	41
Huadu	W35~W53	1.38 (0.65, 2.10)	543 (536, 549)	41
Nansha	W35~W48	0.24 (0.20, 0.28)	476 (465, 487)	41
Zengcheng	W36~W45	0.99 (0.70, 1.28)	339 (331, 347)	41

### Spatial analysis

**Figure 4:**
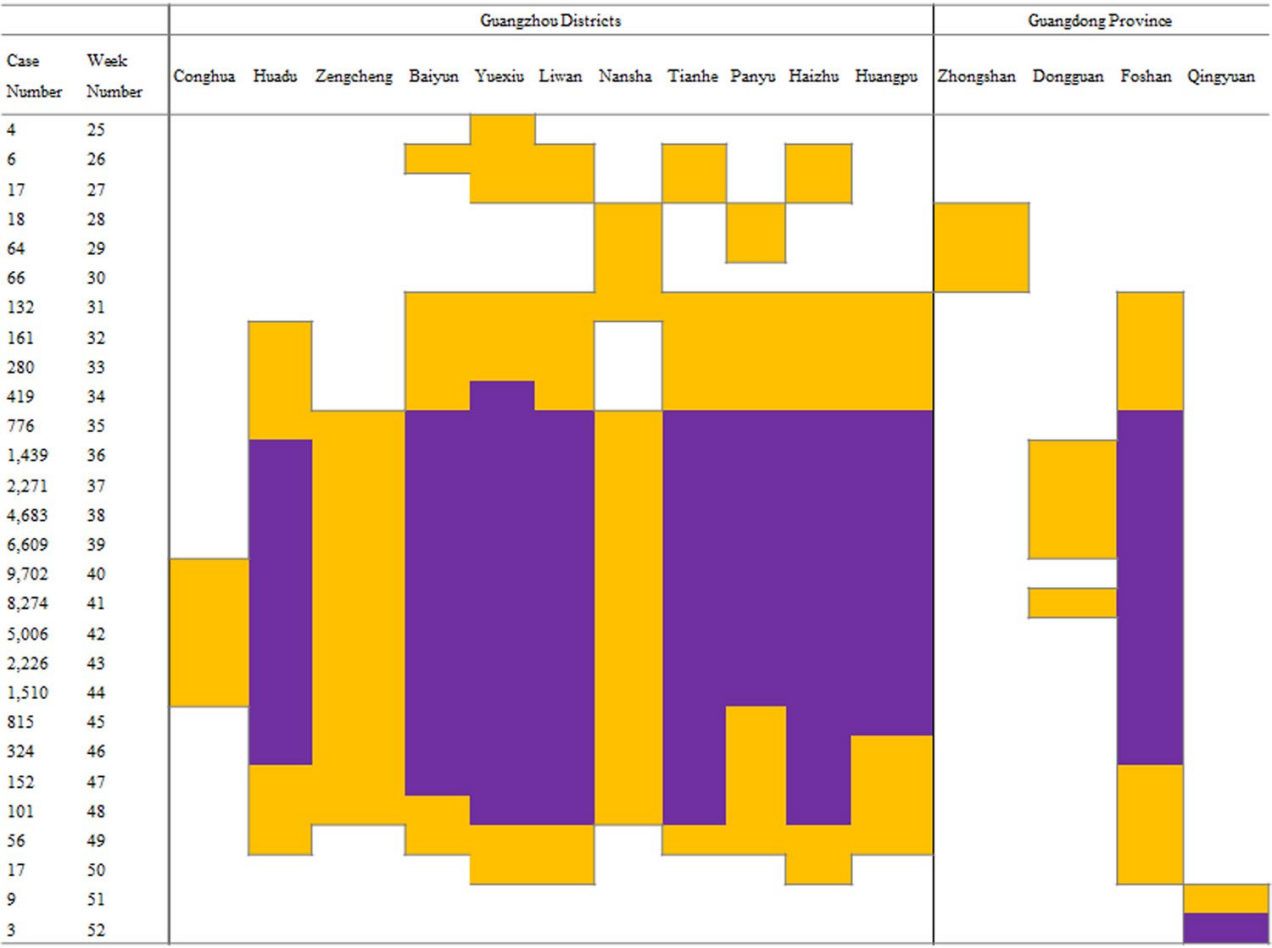
Output of the G* calculations performed on weekly incidence rate data. The first two columns show the weekly number of dengue cases reported in Guangdong, and the week number. The next 11 columns refer to Guangzhou districts, while the last four columns refer to Guangdong cities. Only locations where at least one hot spot is detected are presented. White cells indicate a not significant value of G* (*p*-value > 0.05), while colored cells indicate a significant value of G* at the 0.05 level (orange), and 0.001 level (purple), respectively.

We performed a spatial association analysis, by applying the G* statistic (Fig. 4), which provided insights into the spatial aspects of the spread of the 2014 epidemic in Guangdong. Since Yuexiu district is the only hot spot in week 25, it is reasonable to infer that the epidemic starts around that area. In the following two weeks, it spreads towards all the districts adjoining with Yuexiu, namely Baiyun, Liwan, Tianhe, and Haizhu, but is still confined around the Guangzhou city area. From week 28 to week 30, a shift toward south can be observed, as spatial aggregations of high values of incidence rate are detected around Zhongshan city, and around the districts of Panyu and Nansha. Starting from week 31, the epidemic moves back to north/north-east, and it concentrates around Foshan city, Huadu, Baiyun, Yuexiu, Liwan, Tianhe, Panyu, Haizhu, and Huangpu district. In week 35, there is a further extension to the districts of Zengcheng and Nansha, and from week 36 to 39, a hot spot is identified even in Dongguan. Conghua is the latest district to be classified as a hot spot (weeks 40–44). From the beginning of September to the beginning of November (weeks 36–44), the total number of weekly reported cases in Guangdong province is constantly greater than one thousand. During the same period, a very strong spatial association of high values of weekly incidence rate is detected around Foshan city and around the districts of Huadu, Baiyun, Yuexiu, Liwan, Tianhe, Panyu, Haizhu, and Huangpu (*p*-value < 0.001). Moreover, the same regions are identified as hot spots uninterruptedly from week 32 to week 49, thus indicating them as the core areas of the 2014 outbreak. In the end, the tail of the epidemic is characterized by four hot spots in week 50 (Yuexiu, Liwan, Haizhu, and Foshan) and one hot spot in week 51 and 52 (Qingyuan).

**Figure 5:**
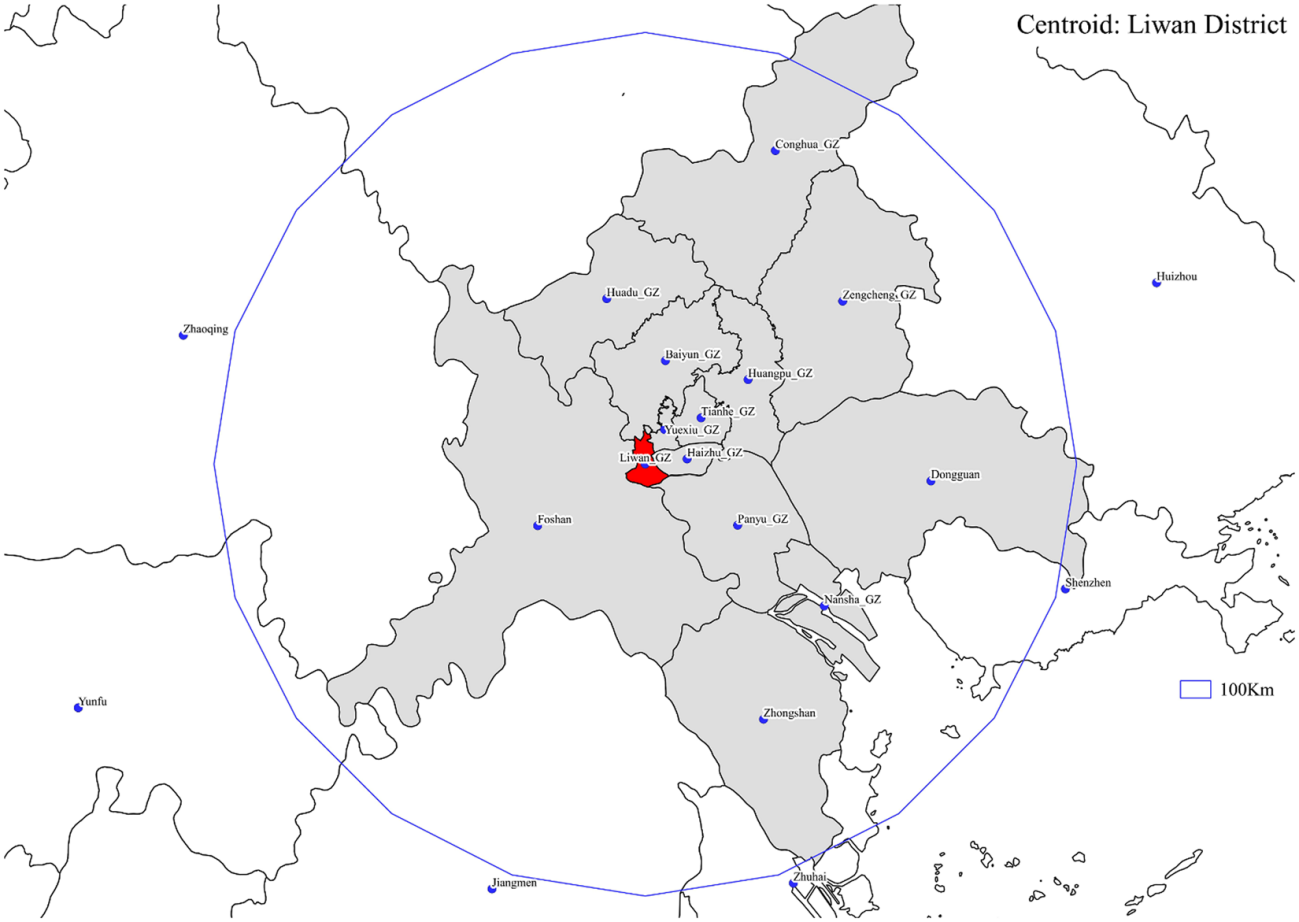
Regions (in grey) falling within 100 Km from Liwan District (in red). Figure 5 was created with the Open Source software QGIS (version 2.16.3 - http://www.qgis.org/en/site/).

To help elucidate the occurrence of the spatial-temporal events, we summarize the chronology in Table 3.

**Table 3:** Summary table for chronology of spatial-temporal events during week 25–52 of the 2014 dengue outbreak in Guangdong province.

Week	Location	Event
25	Yuexiu	First hot spots
26–27	Baiyun, Liwan, Tianhe, Haizhu	More hot spots
28–30	Zhongshan city, Panyu, Nansha	Hot spots moving southward
31–34	Foshan city, Huadu, Huangpu	Disease spreading in the area
35	Zengcheng	Hot spot
36–39	Dongguan city	Hot spot
40–44	Conghua	Last hot spot in Guangzhou
32–49	Foshan city, Huadu, Baiyun, Yuexiu, Liwan, Tianhe, Panyu, Haizhu, Huangpu	Strong spatial association of high values of weekly incidence rate (*p*-value < 0.001)
50	Yuexiu, Liwan, Haizhu, Foshan city	Only four hot spots remaining
51–52	Qingyuan	Only remaining hot spot

### Video slideshow

In order to enhance the information content of Fig. 4, we combined into a video slideshow (Video 1), the results provided by the Richards model fitting and by the spatial association analysis. Video 1 includes 28 maps, simultaneously displaying, for each week, the ongoing waves of cases (cities/districts areas shaded in a color gradient from white to bright red), the occurrence of the turning point (cities/districts areas colored in bright red), and the cities/districts identified as hot spots (centroids in blue). For simplicity’s sake, in Video 1 only the first (0.05) of the two above-mentioned significance levels is used to pinpoint a hot spot.

During the initial stages of the outbreak (from week 25 to week 30, when less than 100 cases per week are reported), both the infection waves and the hot spots are located in a region encompassed between Baiyun district and Zhongshan city. The Richards model also pinpoints a wave to start on week 25 in Foshan city. However, there is no hot spot in this location until week 31. As a contrast, it is interesting to observe that Nansha district is detected to be a hot spot from week 28 to week 31, but, according to the Richards model, the starting time of its wave is four weeks later (week 35). As of week 34, two new waves of infections start in the cities of Jiangmen and Qingyuan, lasting 15 and 21 weeks, respectively. However, Jiangmen was never a hot spot and Qingyuan was a hot spot only for the last two weeks. In weeks 36–44, the weekly numbers of cases were the highest recorded during the epidemic, peaking on week 40 (9,702 cases) and 41 (8,274 cases). The Richards model captures such behavior, by simulating new waves starting in Zhuhai, Maoming, Shenzhen, Dongguan, Zhaoqing and Chaozhou, and by estimating a turning point at week 40 or 41 for almost all the cities/districts (Fig. 3). During the same period, hot spots are detected exclusively in some Guangzhou districts, namely, in Foshan and Dongguan. Moreover, the G* values do not noticeably change up to week 49, when, on the other hand, the epidemic is everywhere in its descending phase, and dengue waves are estimated to be already over in nine localities.

## Discussion

In this study, the combined visualization of Richards model results and spatial association analysis, allows a more comprehensive assessment of the spatial-temporal evolution of the 2014 dengue outbreak in Guangdong. Our integrated approach enables us, as a first step, to determine the turning points of the outbreak, which have obvious epidemiological importance, indicating either the beginning (i.e., moment of acceleration after deceleration) or the end (i.e., moment of deceleration after acceleration) of a wave of infections. We note that the turning points in 2014 differed from city to city, being reached earlier in Guangzhou and its nearby cities. The outbreak spreads initially from the central districts of Guangzhou city, which comprise densely populated areas and work offices where people commute daily and hence are important in driving the epidemic. It then spread over most of the central/western half of the province, probably affected or promoted by factors such as population movement and density, mosquito movement, and climate conditions. The only exception is a wave of cases observed in Chaozhou city, at the eastern border of Guangdong province, perhaps indicating less favorable conditions, less interaction with the disease epicenter of that region, and/or implementation of more effective control measures. Subsequently, there was very little pre-existing anti-DENV immunity among the population in Chaozhou for this epidemic, although 24 cases have occurred in patients infected with DENV-1 in a background of immunity from 2008 epidemic^21^.

The second step, i.e. the spatial association analysis, reveals that, in terms of weekly incidence rate, hot spots are mainly located in Foshan and Guangzhou’s districts, depicting a far more limited high-risk area for dengue transmission in Guangdong in 2014. This leads us to conclude additionally, that the outbreaks detected in Maoming, Jiangmen, Zhaoqing, Qingyuan, Zhuhai, and Shenzhen were not only shorter and milder in terms of incidence, but also unable to spread further spatially, as these locations were not hot spots. The same can also be said about Zhongshan after week 30, while Dongguan played a role, at least during week 36 to week 41. Moreover, the considerable distance and multiple cities between Guangzhou and Chaozhou seem to suggest that the wave of infections in Chaozhou may have a different origin, which requires further investigation.

We also note that the DF hot spots are mainly distributed in Guangzhou districts and in the neighboring city of Foshan, while only one hot spot occurred in the early days of the outbreak (week 28–30) in Zhongshan city, where DF outbreak in 2013 was more serious than that of Guangzhou (incidence in 2013:29.1/100,000 versus 10.5/100,000; incidence in 2014: 21.0/100,000 versus 288.9/100,000). Our findings pertaining to the difference in temporal progression of hot spots in Guangzhou and neighboring Zhongshan city suggest that underlying differences in public health intervention measures implemented might have caused the discrepancy in the outbreak outcomes in these two cities. To be more precise: 1) Zhongshan was the most serious DF affected city in 2013, but the predominant genotypes in both 2013 and 2014 were DENV-1 and DENV-3 and they appeared to be autochthonous^22^, hence pre-existing anti-DENV virus antibodies (including inapparent infections) among the population might have decreased the number of cases. 2) Free hospitalization and hospital isolation (in effort to prevent disease transmission to the vectors) of all DF cases fully implemented in Zhongshan, prevented DF patients from transmitting DENV to susceptible populations, showing that this measure is more effective than individual isolation at home and better in controlling the sources of infection. 3) Guangzhou is recognized as a gateway of domestic and foreign exchanges in Southern China, thus is at risk of imported cases, subsequently expanding epidemic foci and even possibly leading to autochthonous outbreak. 4) A previous study also indicated that the occurrence of DF outbreaks in Guangzhou would impact Zhongshan^23^, indirectly implying that Zhongshan city was not the primary occurrence of DF outbreak and lacks the necessary factors (including population movements, mosquito carried DENV, and others)to independently become a lasting hot spot in 2014.

A recent study^24^ proposes that hospital isolation of cases during the early outbreak period in Guangzhou contributed to reduce the velocity of incidence and the geographical diffusion. Although it is unclear how impactful social-distancing to prevent disease transmission in vector-borne diseases can be, as opposed to that of human-to-human infections, our results appear to support this conclusion (see Fig. 4 and Video 1). Moreover, from Tables 1 and 2, the turning points for cities/districts in or around Guangzhou seem to occur earlier, perhaps implying that some effective measures, such as free hospitalization and treatment of dengue patients, implementation of anti-mosquito programs (including clearing the stagnant water at home and reducing larval sources, killing mosquitoes by insecticide and larvicide, and experimentally releasing *Wolbachia*-infected *Ae. aegypti*^25^)and surveillance of suspected imported DF cases, would mitigate the outbreak. Such evidences should be considered when planning future anti-DF measures, and as illustrates by our study, that a detailed spatial-temporal characterization of an outbreak can be helpful in the assessment of possible impact of control strategies.

Furthermore, the turning points in epidemic areas occurred between weeks 40 and 42 in 2014 (between September 28-October 18), regardless of when the outbreak had first emerged in the area, and whether it was a hotspot or not. Thus, it suggests further investigation is needed to clarify if dengue epidemic termination was resulted from any intervention measures (that were implemented mainly to suppress mosquito populations, to isolate severe cases and to enforce home isolation of mild cases), or due to seasonal changes which might have played a role in decreasing mosquito population and subsequently dengue virus. It is likely that both contributed to the ending of outbreak, and being able to quantitative analyze or characterize these two effects (nature and man-made intervention) in vector-borne disease outbreak such as dengue or Zika is undoubtedly a topic for future research.

The spatial-temporal analysis presented in this paper differs from the one by Zhu *et al*.^10^, both in method and scope. First, the temporal modeling approach here is phenomenological and not compartmental. Second, we examine the whole course of the epidemic over the entire Guangdong province from the beginning of the outbreak in Yuexiu in June (week 25) to the end of the year, while Zhu *et al*.^10^ focuses solely on Guangzhou districts, adopting September-November as the study time period (i.e. from the peek to the end of the epidemic). Third, the aim of Zhu *et al*.^10^ is to develop a model for estimating the actual incidence of the outbreak and simulating the number of daytime/nighttime and local/remote infections. On the contrary, our purpose is to combine geo-statistical analysis and mathematical modeling, to produce results strictly based on real epidemic data.

Thanh Toan *et al*.^15^ analyzed the spatial-temporal evolution of dengue fever in Hanoi (Vietnam), by applying logistic regression, spatial autocorrelation analysis, and cluster analysis. Similarly to what we have done, they use at the same time modeling approach and spatial analysis. However, their results were kept separated, while we tried to integrate them for more comprehensive information. Moreover, their analysis counts on a robust historical dataset, but suffers from the complexity highlighted by Louis *et al*.^13^.

The study by Jeefoo *et al*.^26^ on dengue epidemiology in Chachoengsao province, Thailand investigated the spatial distribution of dengue among villages using both local and global indicators of autocorrelation. Their temporal analysis however differs from ours, as they make use of lag-correlation between dengue and climatic data, which had been studied by other authors (e.g., Cheng *et al*.^8^) and therefore is not a focus of our research. The hot spot detection they performed is quite similar to the analysis we carried out in Guangdong, however, they are not able to identify those hot spots that were actually able to spread the disease beyond their borders. To our best knowledge, this is the first attempt of studying as a whole the single outputs (i.e. the waves) produced by the Richards model, by investigating their mutual relationships through the application of a spatial association analysis. The strength of this study lies in the integration of the Richards model, which describes the temporal dynamics of an epidemic outbreak in terms of duration and turning points, with a spatial association analysis, which identifies statistically significant aggregations of high/low values. Our investigation thus enables us to pinpoint waves of infections that actually spread the disease beyond the borders of the regions from where they were generated.

Finally, Wong *et al*.^27^ carry out a national telephone survey of the Malaysian public aged 18–60 years and re-examine the public view of dengue prevention practice in Malaysia. The result indicates that high level of dengue-related knowledge is associated with dengue prevention. Thus, our study provides useful knowledge, such as growth rate, turning point, and hotspot, for public health authorities and the public to better understand the dengue epidemic in Guangdong in real-time, which would be useful for the public to comply and support intervention measures implemented by the government.

There are three main limitations in this study worth noting. 1) The DF data was obtained from National Notifiable Infectious Disease Reporting Information System (NNIDRIS) of the Chinese Center for Disease Control and Prevention (Chinese CDC), but some not diagnosed or mild DF cases might inevitably fail to be reported, and we are unable calculate the missing rate or implement any data correction. 2) During the outbreak, we were unable to perform a real-time investigation on the distance between two concurrent hot spots, which could have resulted in a broader overall infection risk area. 3) The estimates of the spatial-temporal evolution might have been impacted by other factors; for example, vector biology, temperature, or population migration. Since the Richards model cannot explicitly incorporate the impact of such factors on the extrinsic incubation period or on the vector mortality rate, the analysis of the dengue transmission could contain some inaccuracy.

In summary, our results illustrate the potential benefits coming from assessing the DF epidemic status and trends, and the effect of control measures in China, by combining the Richards model and the hot spot analysis method. Further studies are needed to verify the current modeling approach and to include other potential driving factors (weather, financial input, and social factors), with the final aim of developing an effective assessment system to assist disease control and prevention programs for DF.

## Methods

### Epidemiological Data

The raw epidemiological data used in the present research are the daily lab-confirmed dengue indigenous cases reported in Guangdong province, China during the 2014 outbreak, which affected 45,143 individuals. They are available at the NNIDRIS website of the Chinese CDC, and have been anonymized to protect patient privacy and confidentiality^23^. Data are collected systematically and continuously since 2005, when DF became a legally national notifiable infectious disease in China, and individual cases, both clinically diagnosed and lab-confirmed, are required to be reported by doctors within 24 hours of diagnosis. The daily data for each city in Guangdong were preliminarily aggregated by epidemiological week.

Since the dataset shows an enormous concentration of cases in Guangzhou city (82.7% of the total case number), a spatial association analysis performed at city level would have been vulnerable to being excessively affected by such discrepancy, possibly hiding useful information about the spatial pattern of the epidemic. Consequently, indigenous cases observed at district level, were taken into account for Guangzhou city, the dataset eventually including the weekly dengue data reported in the 11 districts of Guangzhou and the data from the remaining 20 cities of Guangdong province.

The resident population for each city and district in 2014 was retrieved from the Statistics Bureau of Guangdong province website (http://www.gdstats.gov.cn/), to compute the weekly incidence rate, as weekly cumulative number of cases per 100,000 inhabitants.

### Mathematical model

The Richards model^20^ is of the form:

1 $$C(t)=K[1+{e}^{-ra(t-{t}_{i}-\frac{{\rm{l}}{\rm{n}}a}{ra})}{]}^{-1/a}.$$

C*(t)* is the weekly cumulative number of laboratory confirmed dengue cases, with t = 0 the beginning week of a wave of confirmed dengue cases. *K* is the total case number over this wave, *r* is the per capita growth rate of the cumulative case number, *a* is the exponent of deviation of the cumulative case curve, and *t*_*i*_ is the week during which a turning point of this wave occurs, which signifies the timing of an upturn or downturn in the rate of increase in the cumulative case number.

The Richards model is a phenomenological model that depicts the temporal growth of outbreak cumulative case number. However, contrary to the more well-known vector-host compartmental models (e.g., Bailey ^28^; Esteva and Vargas ^29^,^30^), it does not describe the actual disease transmission process. For a detailed review of compartmental dengue transmission models, the readers are referred to Andraud *et al*.^31^.

The major usefulness of the Richards model in infectious disease modeling is two-fold. One, to measure the initial growth rate, often used to estimate the all-important basic reproduction number of an outbreak; and two, to detect waves of infections, in order for authority to monitor the temporal progression of an outbreak and its peak (or turning point). In this study, our focus is the temporal-spatial progression of dengue outbreak, instead of disease transmission which often requires more detailed and high quality human and mosquito data. A recent study^32^ reports that, for incidence data with small observations errors, the Richards model yields accurate point estimates for fitting windows up to the epidemic peak and confidence intervals with better coverage. Therefore, the Richards model is more suitably used.

Three model parameters of epidemiological importance are *K*, *r*, and *t*_*i*_, which can be estimated by fitting the Richards model to the time series of cumulative case data of the outbreak, using standard software package with nonlinear least squares (NLS) approximation subroutine, e.g., SAS or MATLAB. See Hsieh and Chen^33^ or Hsieh *et al*.^34^ for applications of the Richards model to past dengue outbreaks in Taiwan and Cuba.

### Spatial association analysis

The analysis of the spatial association of dengue data during the 2014 epidemic in Guangdong was carried out, by computing the standardized G* statistic proposed by Ord and Getis^35^. This statistic measures the degree of spatial autocorrelation of a set of georeferenced data from a “local” perspective^36^, allowing to detect the presence of hot/cold spots, defined as locations around which a clustering of high/low values is observed. As opposed to “global” spatial autocorrelation indices (as for instance Moran’s I^37^), G* returns one value for each location and it has been extensively used to display and investigate the spatial distribution of infectious disease epidemics, including dengue (see e.g. Jeefoo *et al*.^26^, Jeefoo^38^, Khormi and Kumar^39^, Getis *et al*.^40^, Barrera^41^, Lin *et al*.^42^, Tsai *et al*.^43^).

In this study, we calculated G* week by week from the beginning of the epidemic (week 24) to the end of the year (week 52), for each city/district, using the weekly incidence rate as the observed variable. We adopted a weighting scheme with a 100 Km radius cutoff, where the weight *w*_*ij*_ linearly decreases with the Euclidean distance between the geographic centroids of *i* and *j* (*d*_*ij*_), as follows:

2 $${\rm{If}}\,\,{d}_{ij}\le 100\,Km,\,{\rm{then}}\,{w}_{ij}=1-\frac{{d}_{ij}}{100},\,{\rm{else}}\,{w}_{ij}=0.$$

We determined both the centroids’ positions and their mutual distances, by means of the open source geographic information system QGIS (version 2.16.3, http://www.qgis.org/en/site/). An example is provided by Fig. 5, where the regions whose centroids fall within 100 Km from Liwan District, are colored in grey.

The index G* is defined as a standard variate, thus returning a z-score. In this study, in order to differentiate between the aggregation degree of each cluster, we considered two significance levels: 0.05 (equivalent z-score threshold in a two-tailed test = 1.96) and 0.001 (equivalent z-score threshold in a two-tailed test = 3.29).

## Electronic supplementary material

Video Legend

Video 1

S1 Dataset
