# Supplementary material for: Spatial and Temporal Characteristics of 2014 Dengue Outbreak in Guangdong, China
Source: Sci Rep. 2018 Feb 5;8:2344. doi: 10.1038/s41598-018-19168-6 (PMC5799376; doi:10.1038/s41598-018-19168-6)
Supplement: Supplementary file 1 — Video Legend [file 41598_2018_19168_MOESM1_ESM.doc]

**Spatial and Temporal Characteristics of 2014 Dengue Outbreak in Guangdong, China**

Mattia Sanna^1,*^, Jianyong Wu^2,3,4,5,*^, Yanshan Zhu^2,3,4,5^, Zhicong Yang^6^, Jiahai Lu^2,3,4,5,+^, Ying-Hen Hsieh^1,7,+^

^1^ Department of Public Health, China Medical University, Taichung, Taiwan.

^2^ School of Public Health, Sun Yat-Sen University, Guangzhou, China.

^3^ Key Laboratory for Tropical Disease Control of Ministry of Education, Sun Yat-Sen University, Guangzhou, China

^4^ One Health Center of Excellence for Research &Training, Sun Yat-Sen University, Guangzhou, China.

^5^ Zhongshan Research Institute, School of Public Health, Sun Yat-Sen University, Zhongshan, China

^6^ Guangzhou Center for Disease Control and Prevention, Guangzhou, China.

^7^ Center for Infectious Disease Education and Research, China Medical University, Taichung, Taiwan.

^*^ M. S. and J. W. have contributed equally as co-first authors.

^+^Correspondence and requests for materials should be addressed to Y. H. (email: hsieh@mail.cmu.edu.tw) or J. L. (email: lujiahai@mail.sysu.edu.cn).

# Video Legend

Video 1. The video includes 28 maps created with the Open Source software QGIS (version 2.16.3 - http://www.qgis.org/en/site/), and successively assembled into a video with Microsoft PowerPoint 2013, which simultaneously displaying, for each week, the ongoing waves of cases (cities/districts areas shaded in a color gradient from white to bright red), the occurrence of the turning point (cities/districts areas colored in bright red), and the cities/districts identified as hot spots (centroids in blue). Only the first (0.05) of the two significance levels is used to pinpoint a hot spot.
